# Surgical explantation following early transcatheter aortic valve failure: Technical considerations for complex situations

**DOI:** 10.1016/j.xjse.2026.100149

**Published:** 2026-09-21

**Authors:** R. Michael Reul, Alexander P. Nissen, Bradley G. Leshnower

**Affiliations:** a Emory Aortic Center, Division of Cardiothoracic Surgery, Department of Surgery, Emory Healthcare, Atlanta, Ga; b Department of Cardiac Surgery, MedStar Washington Hospital Center, Washington, DC

**Figure:**
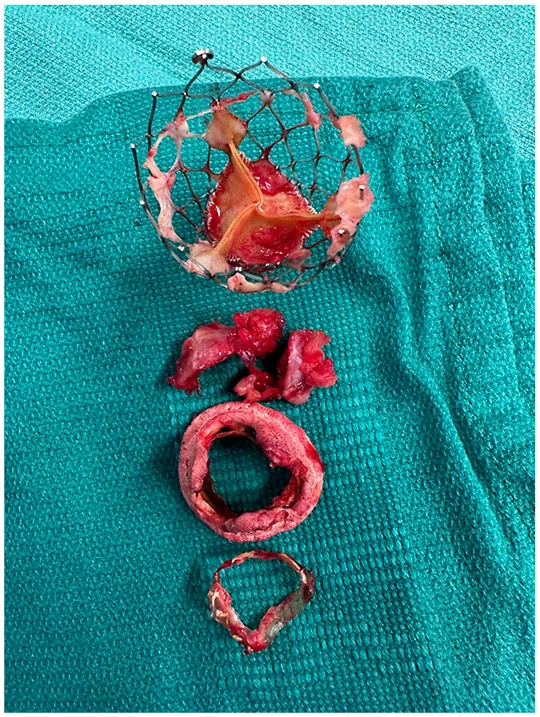
Explanted transcatheter and surgical valves, with early thrombosis and pannus formation.

**Central Message**

Surgical explant of transcatheter aortic valves requires device-specific techniques. Early valve failure and patients undergoing redo sternotomy require additional considerations.

TAVR, transcatheter aortic valve replacement; IRB, institutional review board; LIMA-LAD, left internal mammary artery to left anterior descending; BEV, balloon-expandable valve; SEV, self-expanding valve.

As indications for transcatheter aortic valve replacement (TAVR) expand, the incidence of TAVR failure and complexity of affected patients will continue to increase, compounding already high operative risks.^1^ We present two cases of early TAVR failure following prior sternotomy, representative of the complexity that surgeons will be expected to encounter and manage (Video 1). The Emory University institutional review board (IRB) approved the study protocol and the publication of data (IRB00022795; approved August 3, 2009). Written patient consent was waived by the IRB for publication of deidentified, HIPAA-compliant patient data. Case 1 describes a patient with a patent left internal mammary artery to left anterior descending (LIMA-LAD) graft and early failure of a balloon-expandable valve (BEV) after 21 months (Figure 1). The video depicts considerations for managing LIMA-LAD flow to optimize valve exposure, a double-Kocher technique^2^ for valve removal, and particular considerations for BEVs. Case 2 describes a patient presenting with a stroke 27 months after valve-in-valve TAVR, secondary to severe sinus of Valsalva thrombosis^3^ (Figure 2). The video depicts sharp debridement of the self-expanding valve (SEV) nitinol frame, extensive debridement of the thrombus and pannus ingrowth, and a tourniquet technique^4^ for SEV explantation. We also describe other technical pearls for safe TAVR explant, including preparedness for aortic root replacement and concomitant procedures in these settings.^5^ These cases are presented within an instructional video focusing on techniques and considerations to address complex redo operations requiring TAVR explant.

**Figure 1:**
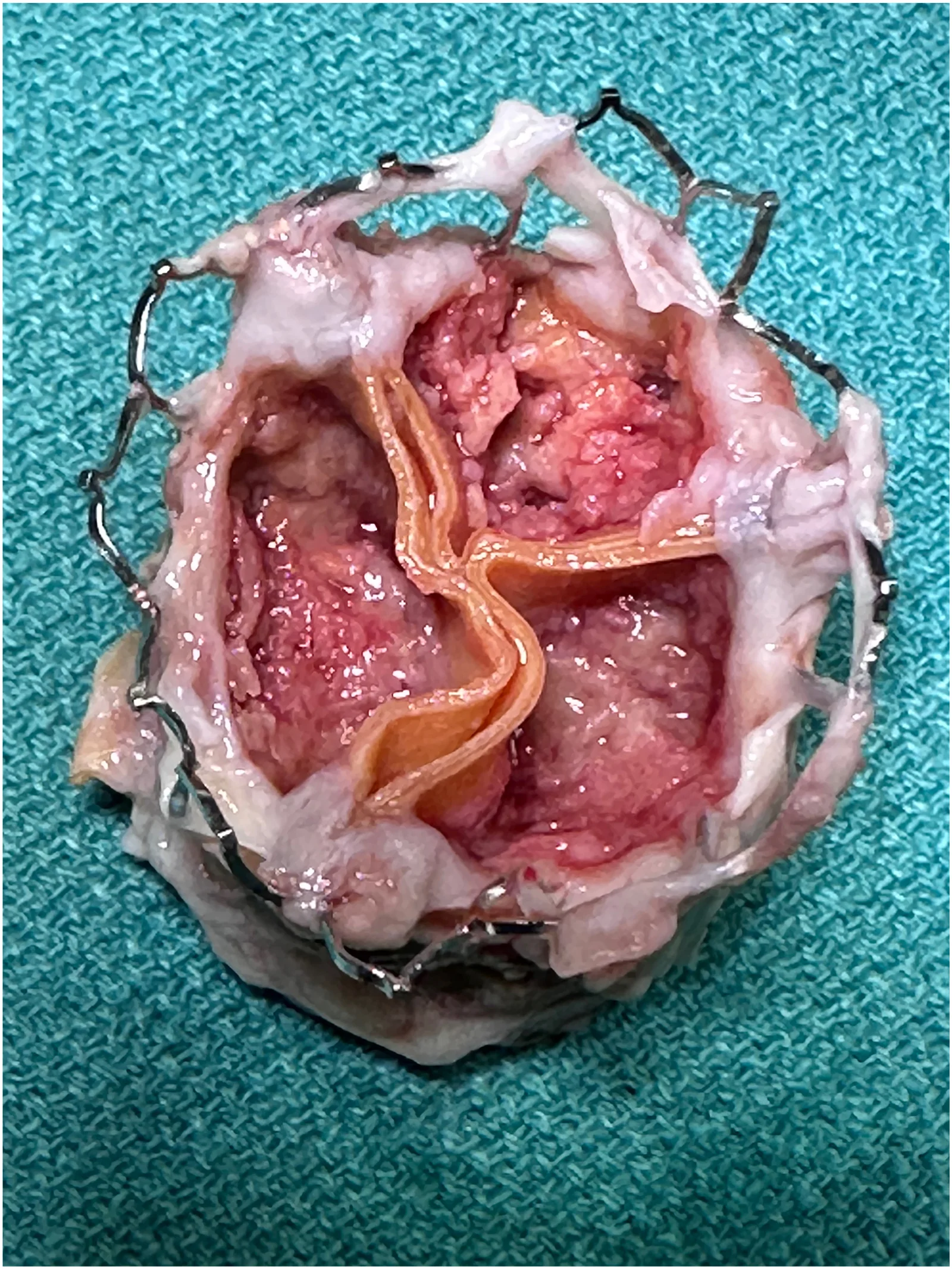
Case 1 surgical specimen. Balloon-expandable valve explant with leaflet pinwheeling, leaflet thickening, and extensive structural valve deterioration.

**Figure 2:**
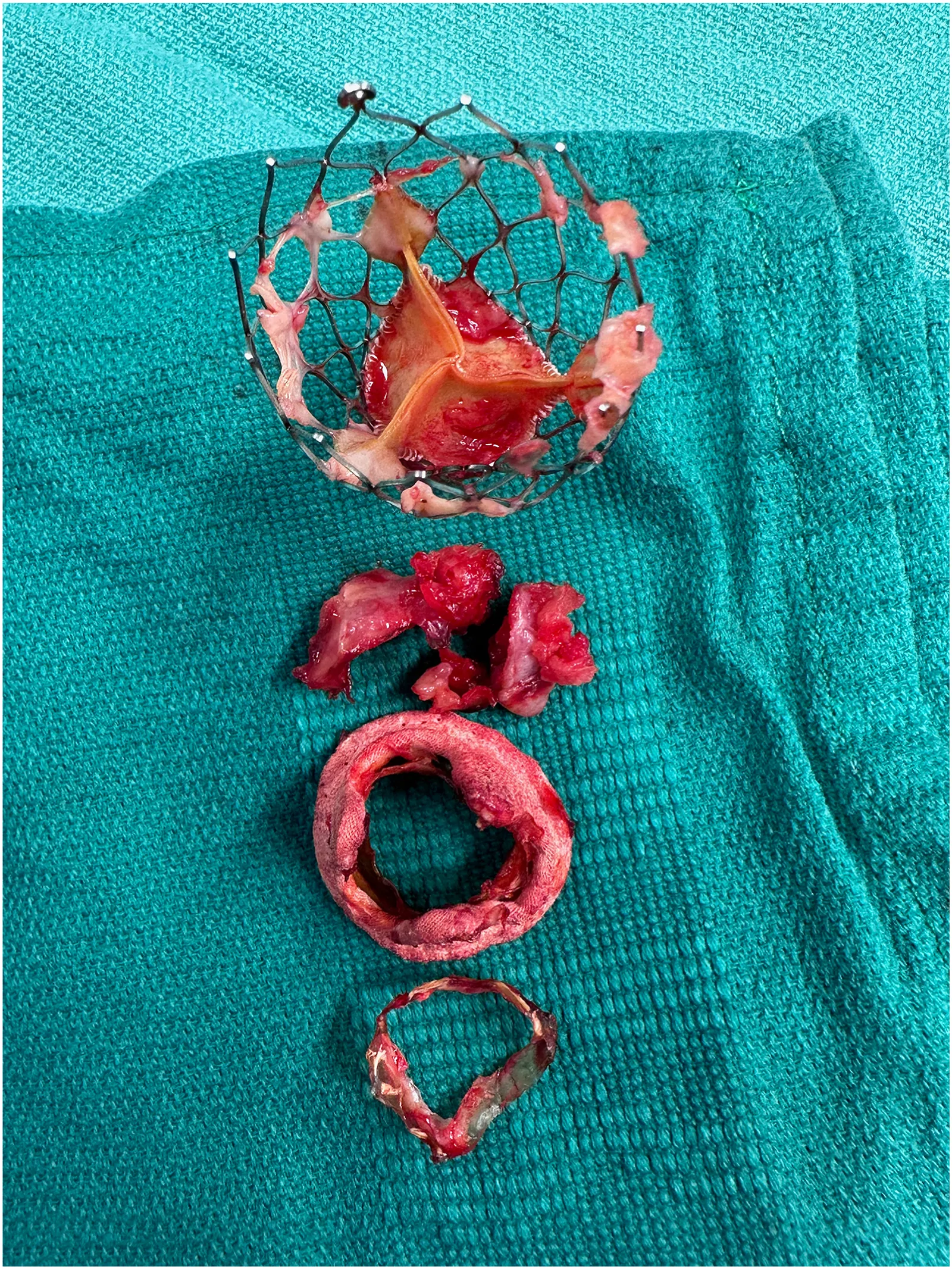
Case 2 surgical specimens. Self-expanding valve with intimal hyperplasia within a nitinol frame and leaflet thickening. Explanted sinus of Valsalva thrombosis. Prior surgical aortic valve replacement. Subannular pannus ingrowth.

### Ethics Declaration

Verbal informed consent to take part in the study and to publish the article was obtained from all participants or their legal representatives. Covered by an IRB, with HIPAA authorization waived. The privacy rights of the participants have been protected.

This study was performed in compliance with relevant laws, regulatory frameworks, and guidelines where the research took place. Ethics committee approval was not required under relevant laws and institutional guidelines. Covered by an IRB, with HIPAA authorization waived.

This research follows the CARE guidelines and the CARE checklist.

## Conflict of Interest Statement

The authors reported no conflicts of interest.

The *Journal* policy requires editors and reviewers to disclose conflicts of interest and to decline handling or reviewing manuscripts for which they may have a conflict of interest. The editors and reviewers of this article have no conflicts of interest.

## Supplementary Data

Video 1 A demonstration of TAVR explant techniques. Case 1 involves management of a patent LIMA-LAD, followed by double-Kocher technique for BEV explant. Case 2 involves extensive sinus of Valsalva thrombosis and root destruction. A tourniquet technique was used, and a root replacement was required.
